# Multimodality Imaging for Transcatheter Tricuspid Regurgitation Interventions: Novel Approaches to the Forgotten Valve

**DOI:** 10.1111/echo.70044

**Published:** 2024-11-29

**Authors:** Francesca Coraducci, Alessandro Barbarossa, Carla Lofiego, Fabio Vagnarelli, Nicolo Schicchi, Marco Fogante, Tommaso Piva, Filippo Capestro, Michela Casella, Marco Di Eusanio, Federico Guerra, Antonio Dello Russo

**Affiliations:** ^1^ Department of Biomedical Sciences and Public Health Marche Polytechnic University Ancona Italy; ^2^ Cardiology and Arrhythmology Clinic Department of Cardiovascular Sciences Azienda Ospedaliero Universitaria delle Marche Ancona Italy; ^3^ Department of Cardiovascular Sciences Cardiology Division “G.M. Lancisi Hospital” Azienda Ospedaliero Universitaria delle Marche Ancona Italy; ^4^ Maternal‐Child, Senological Cardiological Radiology and Outpatient Ultrasound ‐ Department of Radiology Azienda Ospedaliero Universitaria delle Marche Ancona Italy; ^5^ Interventional and Structural Cardiology ‐ Department of Cardiovascular Sciences Azienda Ospedaliero Universitaria delle Marche Ancona Italy; ^6^ Cardiac Surgery Unit ‐ Department of Cardiovascular Sciences Azienda Ospedaliero Universitaria delle Marche Ancona Italy; ^7^ Department of Clinical Special and Dental Sciences Marche Polytechnic University Ancona Italy

**Keywords:** cardiac computed tomography, cardiac magnetic resonance, echocardiography, multimodality imaging, tricuspid regurgitation, tricuspid valve, transcatheter interventions

## Abstract

Tricuspid regurgitation (TR) poses a significant healthcare burden and is a major concern for patients who experience debilitating symptoms and face a poorer prognosis. Cardiologists are showing renewed interest in TR, as the previous belief that it was merely a bystander of left‐sided heart disease has evolved. As a result, more transcatheter techniques addressing TR are emerging. Although a clear impact on mortality from these transcatheter tricuspid valve interventions (TTVI) has not yet been demonstrated, the improvement in symptoms and quality of life for patients is substantial, leading to increased use of these procedures in clinical practice. In this review, we focus on multimodality imaging as an essential tool for quantifying TR severity, assessing right ventricular (RV) function, understanding the underlying mechanisms, selecting the appropriate intervention, and ensuring thorough and accurate preprocedural planning to minimize complications.

## Graphical abstract

**Central Illustration**: The role of multimodality imaging for transcatheter tricuspid valve interventions (TTVI): echocardiography is used for the evaluation of the severity and mechanism of tricuspid regurgitation (TR), for the assessment of the pulmonary pressure and right ventricular (RV) function and for anatomical feasibility for transcatheter interventions. Cardiac magnetic resonance (CMR) is useful for tissue characterization, precise volume assessment, and flow measurement with phase‐contrast sequences. Cardiac computed tomography is used to study the anatomical relationship between the tricuspid valve (TV) and the surrounding structure, to measure the landing zone for transcatheter tricuspid valve implantation, and to assess the anatomy of the caval veins system.

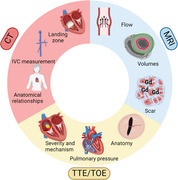

## 1 Why Tricuspid Regurgitation?

Nina Starr‐Braunwald was the first woman to perform open heart surgery, and the first heart surgeon ever to perform an artificial mitral valve replacement (MVR) with a valve she invented [1]. Among all the achievements for which she is known nowadays, there is also a minor paper that she, John Ross, and Andrew Morrow published in 1967 on conservative management of tricuspid regurgitation (TR) in patients undergoing MVR.

They followed up with 28 patients who underwent MVR (8 for mitral stenosis and 20 for mitral regurgitation) with concomitant TR. Of these 28 patients, four died and three of them were also treated with tricuspid annuloplasty. At 30 months follow‐up mean right atrial pressure was consistently lower in all of the patients and all of them showed clinical improvements, therefore the authors concluded that “in advanced mitral valve disease [. .] tricuspid regurgitation is of a functional nature […] and in such patients, tricuspid regurgitation will improve or disappear after mitral replacement and tricuspid valve (TV) replacement is seldom necessary” [2].

This paper set the foundation for understanding TR and its physiological and pathological mechanisms for many years. It was not until the early 2000s, with Nath et al.’s work, that cardiologists began to view the TV as more than just a secondary effect of left‐sided pathologies [3]. Nowadays, the so‐called “forgotten valve” has moved a lot forward, and it is the center of renovated interest among the cardiology community that has now evolved into an “unforgettable” valve.

The interest in TR is sparked due to the increasing evidence that this condition is associated with an increased mortality risk. The meta‐analysis of Wang et al. was one of the first to establish this connection: it showed that moderate to severe TR was associated with twofold increased mortality when compared to no TR even when systolic pulmonary pressure (sPAP) and right ventricular (RV) dysfunction were taken into account [4].

On the other side, it is widely known that a vast majority of TR patients have a concomitant left heart valve disease (around 60%, according to observational data) [5], however, an interesting study from the UK Biobank shows how TR has higher all‐cause mortality than aortic or mitral valve disease [6]. Moreover, data from the European Society of Cardiology Observational Research Programme on Valvular Heart Disease (EORP VHD II) showed that patients with mitral valve disease had a high prevalence of TR, and among these patients, survival is lower for different degrees of TR [7].

The link between prognosis in heart failure (HF) and the degree of TR is well established [8]. Data from a large cohort of HF patients indicates that severe secondary TR was more prevalent among the patients with HF with reduced ejection fraction (HFrEF) but mortality increased for moderate to severe TR in a similar trend across all the HF subtypes [9]. Moreover, in a subanalysis of the GALACTIC‐HF trial, the presence of moderate to severe TR in symptomatic HFrEF patients was independently associated with the primary endpoint of cardiovascular death or HF event with an adjusted HR of 1.12 (95% CI: 1.01–1.26) [10].

More recent data are exploring the connection between HF with preserved ejection fraction (HFpEF) and TR. A recent analysis showed that patients with HFpEF had a higher risk of isolated TR (OR: 1.93) while only half the risk of isolated MR (OR: 0.42) and that isolated TR was independently associated with a worse prognosis [11]. Moreover, the Euro‐TR registry for patients with TR who underwent percutaneous treatment showed that three out of four had a normal left ventricular ejection fraction and two‐thirds of them had increased pulmonary capillary wedge pressures (PCWP), suggestive of HFpEF [12].

Interestingly, the link between HFpEF and TR may also be of a causative nature. A recent study analyzed 30 482 patients with isolated functional TR (FTR) without chronic atrial fibrillation (AF), significant left‐sided pathologies, connective tissue diseases, implantable cardiac devices (CIEDs), or primary lung diseases and found that the prevalence of severe left ventricular diastolic dysfunction was 13% among severe FTR patients. Nonetheless, the probability of HFpEF using the H_2_FPEF score [13] increased with each degree of TR, reaching 88% in severe FTR, alongside an impairment in left atrial strain that was found in almost 70% of patients with severe TR. The authors concluded that “isolated” FTR may not be really “isolated” but is strongly tied with diastolic dysfunction and HFpEF of which FTR could also be the first manifestation [14]. Several mechanisms explain this close relationship: in HFpEF, it is well‐established that over time the right ventricle enlarges, leading to a reduction in fractional area change and subsequent TR [15]. Additionally, HFpEF is closely associated with AF and atrial cardiomyopathy [16, 17].

## 2 Why Transcatheter?

TR surgery is often concomitant with the treatment of left‐sided valve disease, as surgery for isolated TR has historically been associated with a high risk of mortality [18]. American data on 5000 isolated TV procedures reported an in‐hospital mortality of 8.8%, higher for TV replacement than TV repair (with an OR 1,9) [19, 20]. However, newer data showed how in hospital mortality was higher in patients with more advanced stage of the disease and that an earlier referral was associated with shorter postoperative in hospital stay and lower mortality [21]. On the interventional side so far, randomized trials failed to demonstrate a reduction in mortality after treatment of TR. The TRILUMINATE pivotal trial enrolled 350 patients with severe TR at high surgical risk randomized to tricuspid transcatheter edge‐to‐edge repair (T‐TEER) or medical therapy [22]. The mean age of the patient was 78 years and 59% of them was woman. In this pivotal trial the primary composite endpoint was met but the results were primarily driven by improvements in quality of life associated with TR; this results were also confirmed at 1 year analysis of the full randomized cohort (572 patients) [22, 23]. Similar data were corroborated from the bRIGHT registry [24]. Although no mortality benefit was observed, it is important to recognize that in a cohort of elderly and high‐risk patients achieving a reduction in TR and improving quality of life is a significant outcome.

Moreover, the latest 3‐year follow‐up data from the single‐arm TRILUMINATE trial showed that the reduction of TR to moderate or less was persistent over 3 years. Additionally, although this data lacks a control arm, the heart failure hospitalization rate decreased by 75% 3 years after T‐TEER compared to before the procedure, and patients who achieved moderate or less TR were significantly less likely to be readmitted for heart failure (HR 0.48, CI 0.25–0.93, *p* = 0.026) [25]. However, choosing the type of treatment is not a black‐or‐white decision; it involves many grey areas where a careful risk‐benefit analysis is essential.

The traditional paradigm of choosing “old and high risk—transcatheter” versus “young and low risk—surgical” should be reconsidered in light of the new data from the TRIGISTRY [26], where Dreyfus et al. applied the TRI‐SCORE (a multiparametric score including both clinical and imaging parameter that has proven effective in predicting mortality after isolated TV surgery [27]) to a wide cohort of patients with severe FTR treated in different ways: conservative with medical management, surgical repair or replacement and transcatheter repair, followed up for 2 years [26]. In patients with low TRI‐SCORE, there was a significant mortality benefit with both surgical and interventional treatment compared to conservative treatment, moreover, the mortality rate for surgical intervention in low TRI‐SCORE patients was way lower than what previously reported (2.7% vs. around 10%), with a possible explanation for this being that mortality outcomes are not driven by the complexity of the surgery itself but by how far the consequences of severe TR have gone, and by other words, by a late referral to the surgeon [19], as TR is a chronic condition that may give symptoms in an advanced status that is beyond the point where avoiding an irreversible remodeling is possible [11].

Another important data evicted from the TRIGISTRY analysis based on TRI‐SCORE is that survival curves were similar between surgery and the subgroup of patients with successful transcatheter correction and that mortality benefit of successful transcatheter correction of TR reached statistical significance in intermediate‐risk patients when compared to medical treatment. This is an important finding, suggesting that the method of achieving TR correction may be less important than ensuring it is achieved, and widens the indication of transcatheter procedures for TR [26].

Considering all this data together, it is hard to pinpoint a precise timing or type of intervention, as TR is a multifaceted condition that often overlaps with other pathologies, however, lately, the paradigm is shifting from a “wait and see” to “intervene before it's too late” approach.

Despite extensive efforts made to have a complete picture of the effect of TR, there is not yet clear evidence if TR is, besides associated with worse outcomes, a therapeutical target per se rather than an epiphenomenon of a more complex disease that cannot be addressed by treating the regurgitation alone.

## 3 Anatomy, Mechanisms, and Quantification of TR

Before we dive into the complex world of transcatheter tricuspid valve intervention (TTVI) options for TR treatment, it is good to establish some cornerstone concepts.

The TV is the most apical and large valve in the heart, with usually three leaflets (named septal, anterior, and posterior) that can vary in number and size. It has two papillary muscles, the anterior one, larger and attached to the lateral wall of the RV, and a posterior one, that can also be multiple. The anterior papillary muscle supports with chordae the anterior and posterior leaflets and the posterior papillary muscles support the posterior leaflet and the posterior part of the septal leaflet. The anterior part of the septal leaflet as well as the adjacent segments to the anterior and posterior leaflets have chordae that are attached directly to the septum or to a small septal papillary muscle that supports the antero‐septal commissure called the Lancisi papillary muscle [28, 29]. This is a unique feature of the TV apparatus that gives the valve particular sensitivity to changes in RV anatomy and conformation.

The tricuspid anulus is a very peculiar structure and it is very different from its left‐side neighbor. It can be divided into two parts that are very different in role and tissue composition [30]. The largest part is the “mural” portion that arises from the enfolding of the atrial wall and the ventricular wall into the atrioventricular groove. This part is characterized by a scarce presence of fibrous tissue and a larger quota of epicardial adipose tissue covered on the internal side by the endothelium [31]. There are differences in the characteristics of the mural annulus between males and females. In males, there is a greater presence of elastic fibers, whereas in females, the annulus is larger even when adjusted for body surface area [32, 33]. On the other side, the septal anulus is made by the hinge line of the septal leaflets to the interventricular septum, due to the proximity with the membranous septum, this part of the anulus is reinforced by connective tissue fibers.

**FIGURE 1:**
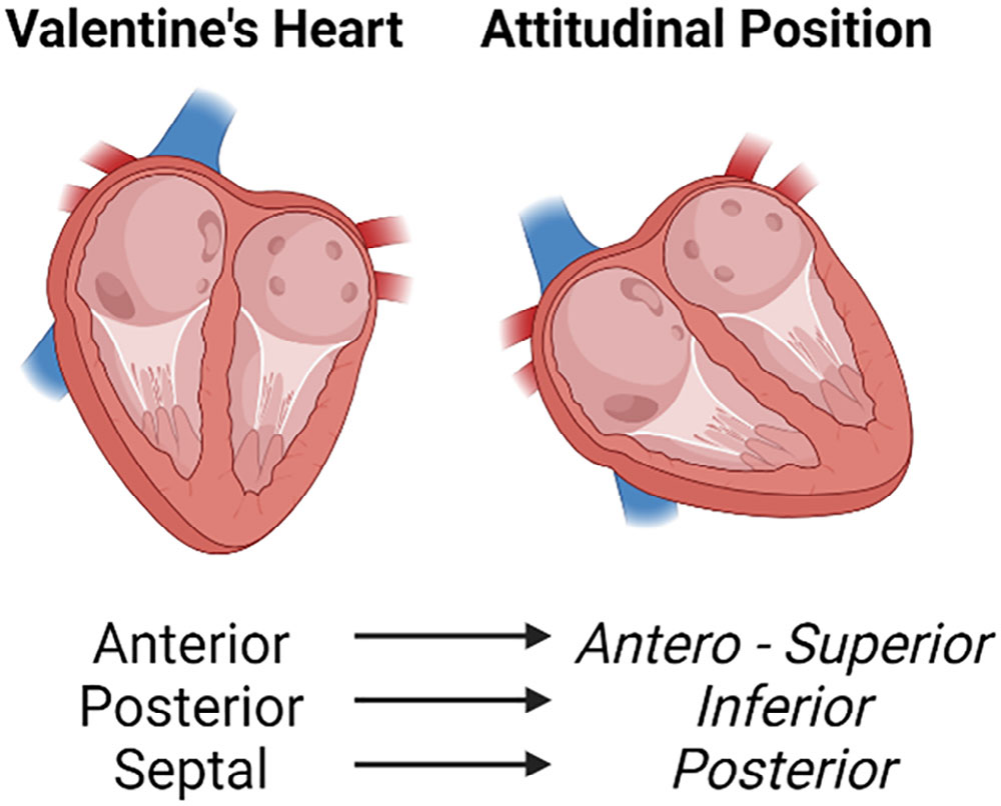
On the right: the heart pictured in a vertical positioning as a “Valentine's heart,” on the left: the heart pictured with the long axis rotated to the real “apex” position. The “anterior” leaflet is truly located antero‐superior, the “septal” leaflet is posterior, and the “posterior” leaflet is inferior.

The anatomy of the TV leaflets is complex and highly variable among patients. The usual nomenclature of the leaflets (anterior, posterior, and septal) refers to the so called *“valentine's heart,”* instead, in the “attitudinal position,” where the long axis of the heart is rotated and points to the real apex of the heart, the anterior leaflet *is antero‐superior*, the septal leaflet is *truly posterior* and the posterior leaflet is *inferior* (Figure 1) [34, 35]. Even though this nomenclature is based on the “surgical” display of the valve, the misleading nature of this naming system must be well clear in mind when coming to interventional procedures as this can cause pitfalls in the communication between the imager and the operator.

Hahn and colleagues developed a new morphological classification for the TV with four subtypes based on the number of leaflets: type I with three leaflets, type II with two leaflets by fusion of the anterior and posterior leaflets, type III with four leaflets, and type IV with more than four leaflets [36]. Type III is further divided based on the location of the fourth leaflet (IIIA with two anterior leaflets, IIIB with two posterior leaflets and type IIIC with two septal leaflets).

On the other hand, the traditional classification of TR based on the mechanism of regurgitation has been revised lately: primary TR arises from structural alteration of the leaflets (for instance in congenital abnormalities, carcinoid disease, infective endocarditis, and so on), secondary TR is now subdivided into two distinct categories like ventricular TR (V‐TR) and atrial TR (A‐TR). Cardiac implantable electronic device‐related TR is now a distinct category [28, 35].

## 4 Anatomical Considerations for Interventional Procedures

### 4.1 Adjacent Structures

TV has many relationships with adjacent structures, and basic knowledge of those relationships is crucial for procedural success. First, the antero‐septal commissure lies next to the non‐coronary sinus of Valsalva. Although this relationship is an important echocardiographic marker to distinguish the anterior‐septal commissure, risk of aortic perforation must be taken into account when targeting this area. Secondly, the right coronary artery (RCA) runs within the atrio‐ventricular groove with a proximity to the leaflet that can shorten up to 1 mm in the posterior part of the mural anulus. Devices that target that region are at high risk of producing an acute ischemic injury by anchoring the RCA. Third, the atrio‐ventricular node (AVN) is in the Koch's triangle, a structure anatomically determined by the septal annulus, the tendon of Todaro, and the ostium of the coronary sinus. Right below the AVN is the His bundle that lies only 3–5 mm far from the anteroseptal commissure. Therefore, a non‐negligible risk of AV block must be taken into account in all procedures on the TV anulus [29, 37].

### 4.2 TV Anulus

The anulus has a saddle shape configuration and is highly dynamic, with a variation of TV area up to 30% during the cardiac cycle [33]. In vitro models suggest that, due to the larger area compared to the mitral valve, only a 40% increase in the tricuspid anulus may result in significant TR rather than the 75% increase needed for mitral valve regurgitation [38]. Due to its peculiar configuration, in case of chronic AF or atrial cardiomyopathy, annular dilatation can occur but mostly involves the mural part of the anulus with an augmentation of the distance between the septum and the lateral wall. In the presence of significant FTR, Nu et al., demonstrated using 3D echocardiography, how the anulus becomes larger, more planar, and more circular.

### 4.3 Subvalvular Apparatus and RV

In case of a RV dilatation or dysfunction, the papillary muscles are displaced toward the apex, the leaflets are tethered and coaptation is impaired. On the other hand, the risk of RV outflow tract obstruction due to TTVI is minimal due to embryological reasons, as there is a wide separation between the infundibular region with the pulmonary valve and the TV.

## 5 Patient and Device Selection

Before reaching interventions it is mandatory to treat TR with medical therapy. Cornerstone of medical therapy for TR is diuretics to avoid fluid overload [39]. The rise in central venous pressure and volume overload with severe TR can lead to RV remodeling and can also be responsible for diuretic resistance due to “renal tamponade” and slow splanchnic venous flow that reduces drugs adsorption [40, 41]. Second cornerstone of medical therapy for TR is the treatment of AF: it has been reported that those with permanent AF are at higher risk of developing significant TR and that patients who achieve rhythm control have a reduction in TR jet area [42, 43].

In the rarer cases of CIEDs‐induced TR, lead extraction has been reported to be effective in reducing TR, but clinicians should be aware that the risks of damaging the TV with this procedure are not negligible [44, 45].

After optimal medical treatment is up‐titrated to the maximum tolerated dose and rhythm control achieved, TR must be re‐assessed and indication to transcatheter intervention re‐evaluated.

There are different transcatheter strategies to correct TR: (1) direct or indirect annuloplasty, (2) restore leaflet coaptation, (3) orthotopic TV replacement, (4) heterotopic TV implantation (caval devices). Table 1 lists the main available devices for TTVI.

**TABLE 1:** Main TTVI strategies and devices available for clinical use or not yet approved with CE mark.

Strategy	Device	Manufacturer, state	Target	CE mark (y/n)
Annuloplasty	TriCinch	4‐Tech, Ireland	Direct annuloplasty	No
	Trialign	Mitralign, Massachusetts	Bicuspidization of the TV	No
	Cardioband	Edwards Lifesciences, California	Direct annuloplasty	Yes
Restoration of leaflet coaptation				
	FORMA system	Edwards Lifesciences, California	Restoring the leaflet gap with spacer	No
	Triclip	Abbott Vascular, California	TV leaflets clipping	Yes
	PASCAL	Edwards Lifesciences, California	TV leaflets clipping	Yes
	Dragonfly	Venus Medtec, China	TV leaflets clipping	No
Heterotopic TV implantation/caval devices				
	TricValve	Products and Features Vertriebs, Austria	Preventing TR‐induced caval backflow	Yes
	Tricento	NVT GmbH, Germany	Preventing TR‐induced caval backflow	No
	Caval SAPIEN 3	Edwards Lifesciences, California	Preventing TR‐induced caval backflow	No
TV replacement				
	Gate	NaviGate Cardiac Structures, California	TV replacement with prosthetic valve	No
	Evoque	Edwards Lifesciences, California	TV replacement with prosthetic valve	Yes
	LuX‐valve	Ningbo Jenscare Biotechnology Co., China	TV replacement with prosthetic valve	No

Abbreviations: TTVI, transcatheter tricuspid valve interventions; TV, tricuspid valve.

Multimodality imaging is crucial in selecting the best device for the right patient, for preprocedural planning and intra procedural monitoring as well as long‐term follow‐up.

Devices for leaflets approximation are optimal if TR is of a secondary etiology with small septo‐lateral gap (< 7 mm), anteroseptal jet location, confined prolapse or flail, and for some cases of CIEDs‐related TR [46]. Table 2 lists the optimal echocardiographic criteria for T‐TEER.

**TABLE 2:** Optimal or unfavorable anatomical and functional criteria for patient selection for T‐TEER.

	Optimal	Unfavorable
**Anatomy**	Coaptation Gap < 7 mm	Coaptation Gap > 8 mm
	Anteroseptal Jet	Anteroposterior jet
	Tri‐leaflets morphology	Uncommon TV morphology/severe leaflets abnormalities
	Localized prolapse or flail	Multi‐scallop flail/prolapse
	Absence of calcification	Presence of calcification
	Favorable angle	Unfavorable angle
**Function**	Normal RV dysfunction	RV dysfunction
	No to mild RV dilatation	Moderate to severe RV dilatation

Abbreviation: T‐TEER, tricuspid valve transcatheter edge to edge repair.

Transcatheter tricuspid valve replacement (TTVR) with the Evoque Valve (Edwards Lifesciences) has recently been approved for clinical use after the positive results of the TRISCEND trial at 1‐year follow‐up [47]. TTVR is optimal in patients who had a previous repair of the TV or have leaflet thickening, shortening, or have been rejected for T‐TEER due to complex leaflet morphology [48]. Moreover, TTVR can be performed in patients with CIEDs leads, accepting that the risk of jailing and endocarditis is hard to quantify [39, 45]. Caution should be taken to the RV systolic function, as the RV has to compensate for the acute increase in afterload obtained with the reduction of TR, even if acute afterload mismatch after TTVI is rarely reported [49].

Direct or indirect annuloplasty has the highest efficacy if performed on atrial FTR with mild tenting height (< 0.76 cm); instead, for shorter leaflet lengths and for CIEDs‐related TR may not be optimal. The lack of strong connective tissue on the mural anulus represents a concrete risk of dehiscence and of RCA perforation [39, 50]. So far, Cardioband (Edwards Lifesciences) is the only device for annuloplasty with CE mark. Data for the clinical use come from the TRI REPAIR study: single‐arm prospective trial that enrolled thirty patients at high surgical risk with moderate or more TR to percutaneous annuloplasty with Cardioband and found a sustained improvement in KCCQ over 2 years of observation [51].

Lastly, The TricValve system (Products & Features) is the first heterotopic caval valve implantation approved with CE mark. It consists of two stents with bovine pericardial leaflets that are implanted in the caval veins to reduce fluid accumulation and venous backflow. This system was approved after safety and efficacy (in terms of symptoms and quality of life) were proven in a cohort of high‐risk patients with severe TR, severe RV systolic dysfunction, high serum creatinine, and high sPAP at 6 months and 1‐year follow‐up [52, 53].

## 6 Multimodality Imaging Considerations

### 6.1 Transthoracic Echocardiography

Transthoracic echocardiography (TTE) is the primary imaging modality for evaluating TR. It is valuable for the initial assessment of valve morphology, grading TR severity, and evaluating the structure and function of the right heart chambers.

**FIGURE 2:**
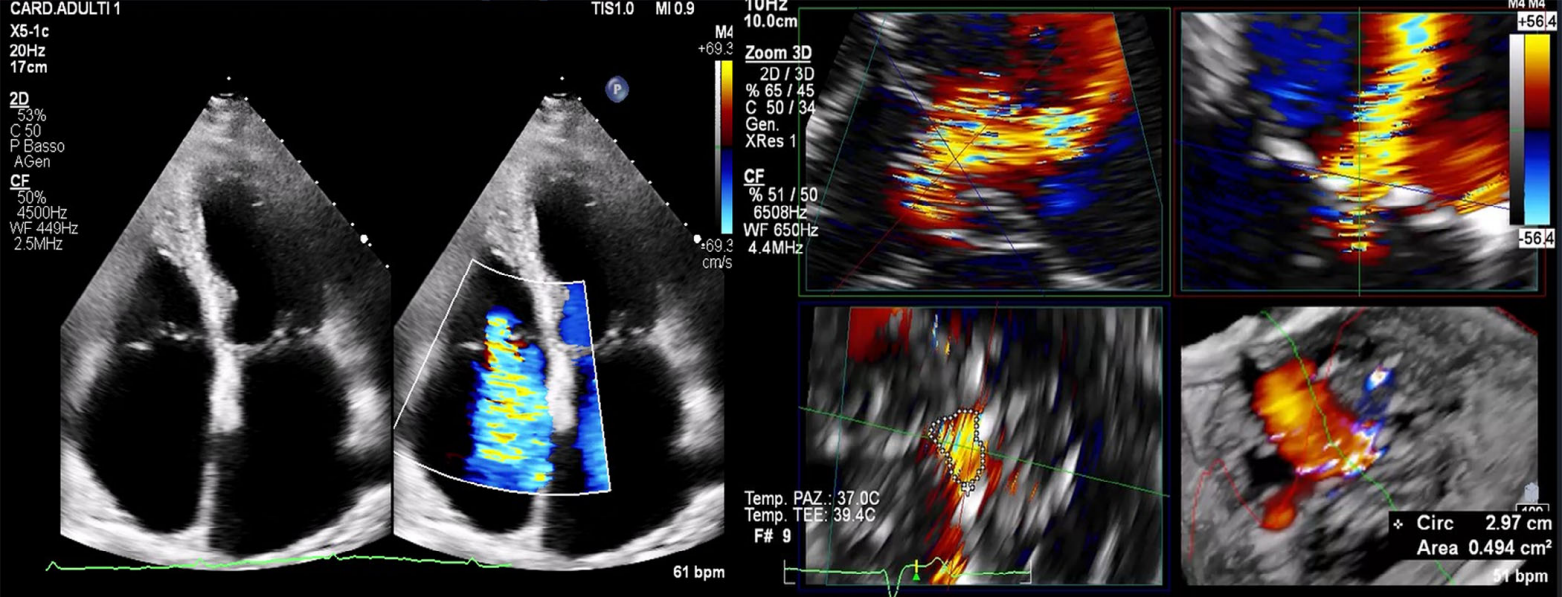
On the left: TTE four chambers view with and without color Doppler, optimal view to measure the 2 dimensional (2D) “VC”; On the right: TOE 3D reconstruction of 3D EROA. 2D indicates 2 dimensional; EROA, effective regurgitant orifice area; TOE, transesophageal echocardiography; TTE indicates transthoracic echocardiography; VC, vena contracta.

Recently, the classification for TR has expanded from a three‐degree to a five‐degree system by adding “massive” and “torrential” categories [35, 54]. This enhanced classification is more sensitive to variations in TR severity after TTVI, as significant clinical improvements can result from modest reductions in TR. In assessing severity, 3D vena contracta has proven to be more accurate than its 2D counterpart and may serve as a reliable alternative to measure the effective regurgitant orifice area (Figure 2) [55, 56].

**FIGURE 3:**
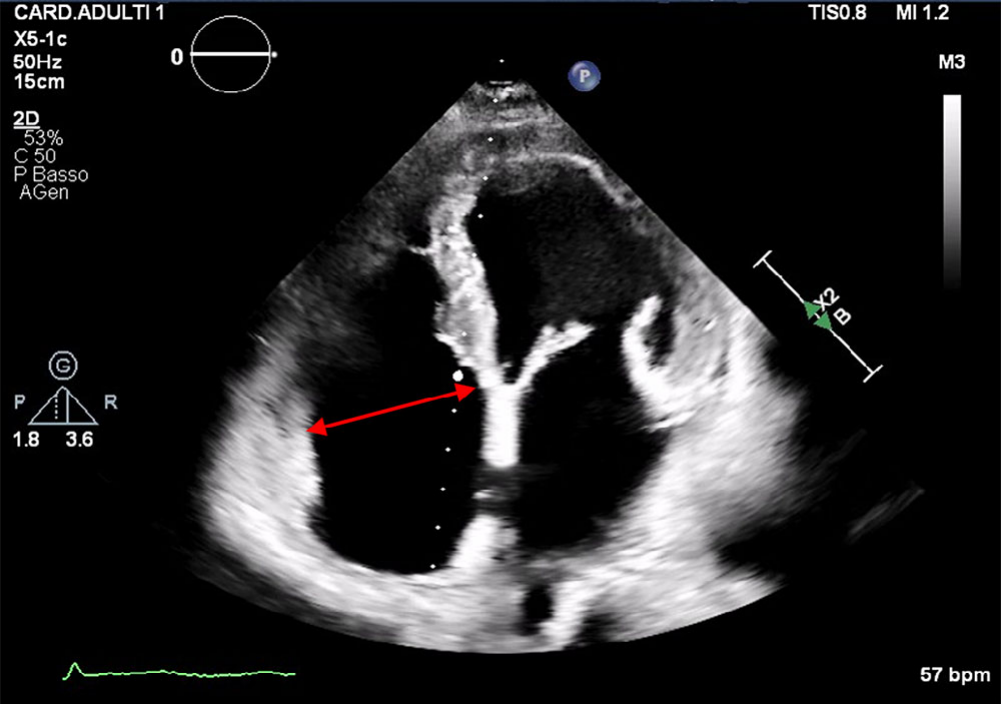
TTE four chambers view at tele‐diastole. Red two‐headed arrow pointing the measurement for the TV anulus. TTE indicates transthoracic echocardiography; TV, tricuspid valve.

TTE is also the reference modality for measuring the TV anulus. As measured in apical four chambers view, normal anulus is 28 ± 5 mm and is defined as dilated if ≥ 40 mm or 21 mm/m^2^ even though increasing evidence suggests the highly variable nature of TV annulus dimensions according to sex, age, and body surface area (Figure 3) [57, 58]. Moreover, analysis from 3D echocardiography suggests that TV anulus is more likely enlarged on the septo‐lateral diameter [59] (along the attachment line of the anterior and posterior leaflets), therefore 2D measurement in apical four chambers view may not be the best way to measure the anulus in this context. No precise cut‐off for 3D measurement of the anulus is available but the 3D measurement is more likely to be larger than 2D [33, 60].

RV dysfunction is a solid contraindication for interventional treatment of TR [61] and TTE for assessing RV systolic function is mandatory prior to TTVI [62], in fact, RV fractional area change < 44% is an independent predictor of survival after T‐TEER [63]. Recently 3D TTE has emerged as a new trustworthy technique to assess RV function and has shown a good correlation with CMR [64].

A deep understanding of the interaction between RV and the pulmonary artery (PA) is also useful for patients undergoing TVVI and indexes estimating RV‐PA coupling are raising more and more interest among echocardiographers. One of the most used is the ratio between tricuspid annulus planar excursion (TAPSE) and sPAP [65]. A TAPSE/sPAP ratio < 0.406 mm/mmHg predicts RV‐PA uncoupling and has proven as an independent predictor of all‐cause mortality after 1 year from TEER in severe TR patients. [66] However, PA pressures estimated by echocardiography in patients with severe TR should be interpreted with caution. In these cases, the accuracy of echocardiography in detecting pulmonary hypertension, when compared to right heart catheterization, is only 55% [67]. This is because the estimated sPAP is based on the peak TR regurgitation velocity, which may be low in severe TR due to rapid pressure equalization between the right atrium and ventricle [68]. Therefore, RHC is often essential in the initial evaluation of patients with TR to overcome the limitations of echocardiographic assessment of RV‐PA coupling. Data from a large retrospective cohort suggest that the TAPSE/mPAP ratio obtained via RHC is a better predictor of 2‐year mortality after TTVI in severe TR patients than the echocardiographic TAPSE/sPAP ratio. Furthermore, the authors were able to establish sex‐specific thresholds for the TAPSE/mPAP ratio to enhance prognostic stratification in these patients [69].

### 6.2 Transesophageal Echocardiography (TOE)

**FIGURE 4:**
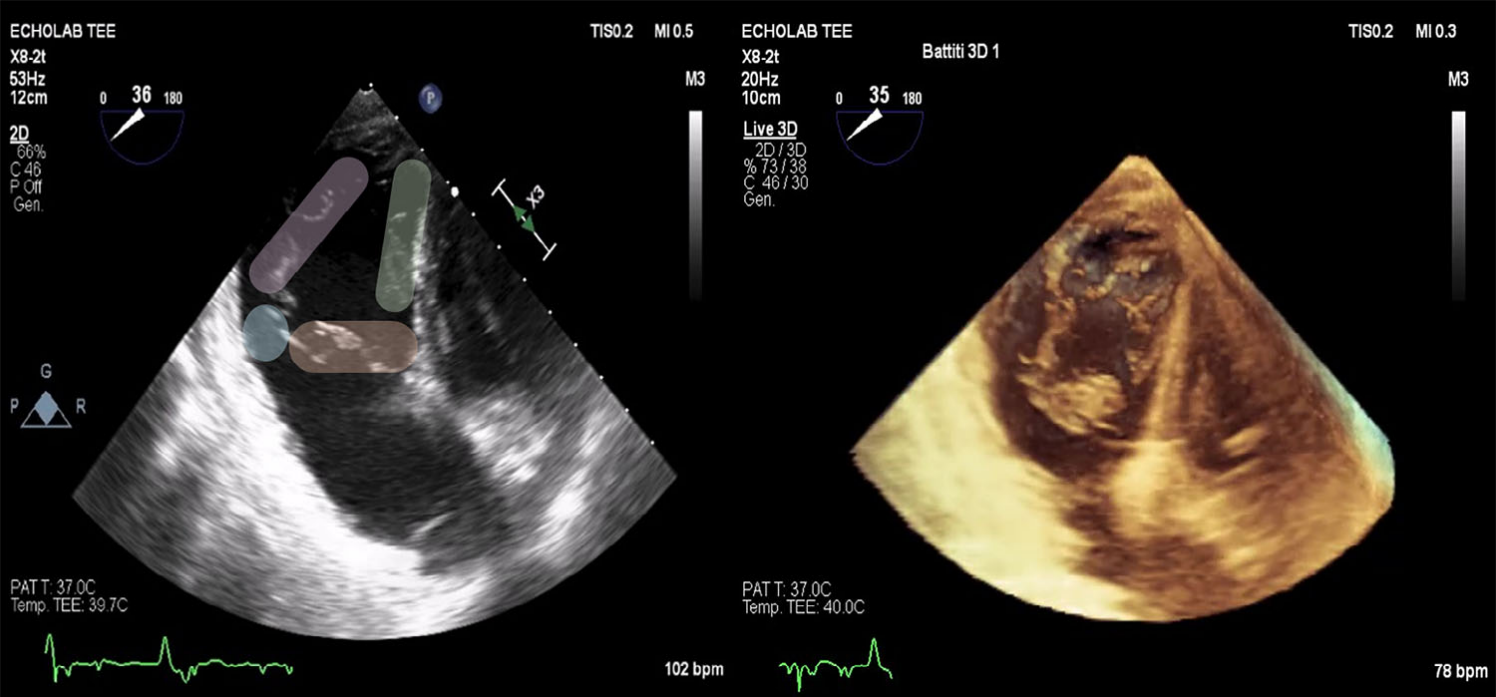
On the left: TOE TG TV oriented view: green oval indicating septal leaflet, purple oval indicating posterior leaflet, orange oval indicating anterior leaflet and blue circle indicating the antero‐septal papillary muscle. On the right: TOE TG TV‐oriented 3‐dimension live view. TG indicates trans gastric; TOE, transesophageal echocardiography; TV, tricuspid‐valve.

**FIGURE 5:**
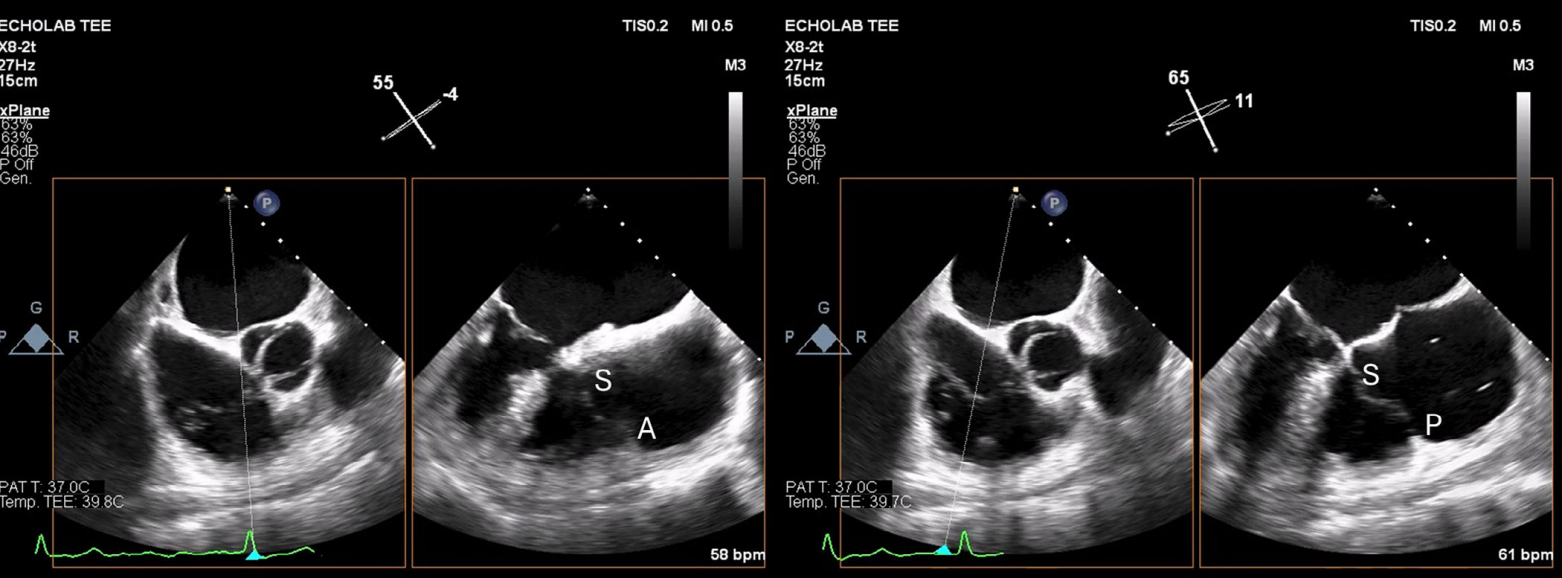
TOE mid‐esophageal 60° RV inflow‐outflow view with simultaneous biplanar imaging. On the left image simultaneous biplanar imaging is positioned to obtain a 90° view for the S and the A. On the right image the simultaneous biplanar imaging is positioned to obtain a 90° view for the S and the P. A indicates anterior leaflet; P, posterior leaflet; RV, right ventricle; S, septal leaflet; TOE, transesophageal echocardiography.

TOE is the key imaging modality for TTVI. It provides a thorough and detailed evaluation of TV morphology (Figures 4 and 5), as well as the severity and underlying mechanism of TR. TOE is crucial in assessing both the anatomical and functional suitability for TTVI, enabling accurate preprocedural planning and appropriate device selection. Optimal TOE parameters for T‐TEER are listed in Table 2 [29]. Moreover, some echocardiographic parameters evaluated during the preprocedural assessment can be used as predictors of TR relapse after correction.

More favorable outcomes after T‐TEER are obtained if the regurgitant jet is located at the anteroseptal commissure [46]. On the other hand, non‐central or non‐anteroseptal jets are independent predictors of poorer procedural outcomes and a higher risk or significant residual TR after the procedure [46].

It is also essential to measure the gap size at the target implantation zone, as it is closely linked to the likelihood of a successful device placement [70].

Data from the TriValve registry that considers different strategies for transcatheter procedures on the TV (anulus correction, leaflet repair or coaptation devices placement, and valve replacement) found out that a greater coaptation depth was an independent predictor of lower procedural success, with an OR of 24.1 [71].

**FIGURE 6:**
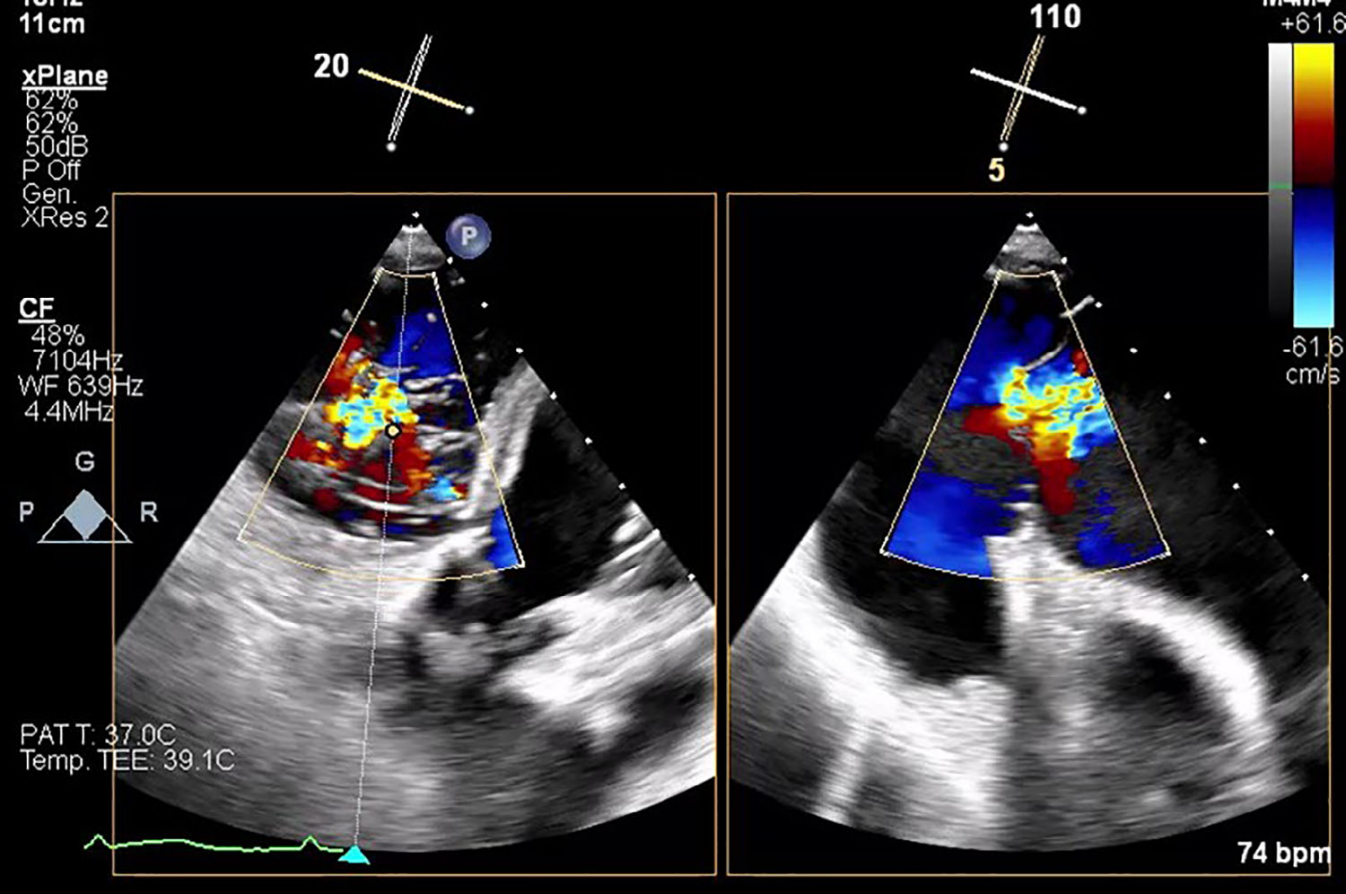
TOE TG view color Doppler and simultaneous biplane imaging that shows a “central” regurgitation (see GLIDE score). TG indicates trans gastric; TOE indicates transesophageal echocardiography.

Recently, Gercek and colleagues developed the interesting “GLIDE Score” to predict postprocedural success in patients who undergo T‐TEER [72]. Procedural success, defined as a postprocedural TR grade moderate or less, was observed in 97% of patients with GLIDE score of 0 or 1 point, 61% of those with GLIDE score of 2 or 3 points, and 14% of those with GLIDE score ≥ 4 points. The score is simple as comprehends only five parameters and is very strong due to the external validation cohort. The score parameters are shown in Table 3 [72]. An example of “central jet” is shown in Figure 6.

**TABLE 3:** GLIDE scoring system.

		0 point	1 point
**G**	Septolateral gap	≤ 5 mm	≥ 6 mm
**L**	Predominant jet location	anteroseptal or central	posteroseptal, anteroposterior or diffuse
**I**	Image quality	Good	Bad
**D**	Chordal structural density	Modest	High
**E**	“En face” TR jet morphology	Oval/linear	Star‐shaped

Abbreviation: TR, tricuspid regurgitation.

Beyond the preprocedural planning, TOE is crucial to guide TTVI in every step of the procedure. Key steps for guiding T‐TEER procedure are listed in Table 4 [62, 73, 74].

**TABLE 4:** Main steps for T‐TEER with the author's suggestion for best imaging TOE visualization.

#	Procedural step	TOE view
1	Guidewiring and delivery system placement in RA	ME bi‐caval view + simultaneous biplane imaging
2	Septal leaflet visualization Clip trajectory	ME inflow outflow view + simultaneous biplane imaging
3	Clip rotation and advancement of the clip in RV	TG SAX + simultaneous biplane imaging
4	Checking proper clip positioning and leaflet grasping	‐ ME RV inflow–outflow view + simultaneous biplane imaging with scanning plane positioned perpendicular to the clip's arms ‐ TV 3D imaging ‐ TG SAX view
5	Checking before releasing	‐ RV inflow–outflow view + simultaneous biplane imaging ‐ TV 3D imaging ‐ TV inflow gradient assessment ‐ Residual TR assessment

Abbreviations: 3D, 3 dimensions; ME, mid esophageal; RA, right atrium; RV, right ventricle; SAX, short axis; TOE, transesophageal; TR, tricuspid regurgitation; T‐TEER, tricuspid valve transcatheter edge to edge repair; TV, tricuspid valve.

### 6.3 Cardiac Computed Tomography

CT plays an important role in TTVI, but when routinely performed without a specific intention to study the TV, the right heart structures are weakly contrasted and the TV is suboptimally visualized [75]. Moreover, most TR patients have AF and particular attention to the heart rate prior CT exam must be taken. Therefore, specific CT protocols for contrast media injection that integrate different variables like weight, left ventricular ejection fraction and heart rate have been suggested [76, 77].

Cardiac CT is crucial in every step of TTVI. First, it can evaluate the access site for percutaneous interventions (particularly for devices requiring large bore access like FORMA, Edwards Lifesciences, Irvine, California or GATE, NaviGate Cardiac Structures, Inc., Laguna Hills, California) [29].

**FIGURE 7:**
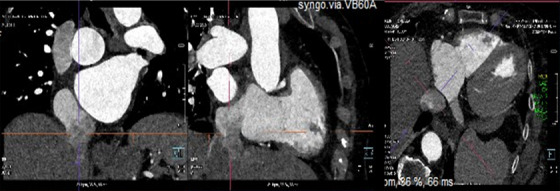
Multi‐plane reconstruction of the IVC to measure the distance from the right atrium and the diameter of the IVC to select the right size for heterotopic TV implantation. IVC indicates inferior vena cava; TV, tricuspid‐valve.

CT is also useful when evaluating the target site, for example in case of heterotopic valve implantation, where is needed for the correct sizing of the cavo‐atrial junction and to avoid hepatic vein occlusion and prevent the systolic backflow of TR (Figure 7) [29, 78].

**FIGURE 8:**
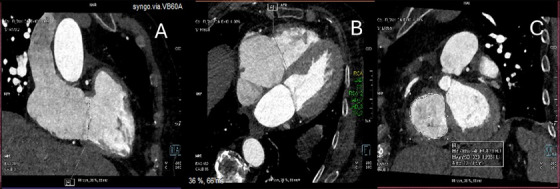
Anulus measurement in CT with multi plane reconstruction. (A) antero‐superior diameter; (B) septo‐lateral diameter; (C) multiplane reconstruction with measurement of the entire anulus. CT indicates computed tomography.

**FIGURE 9:**
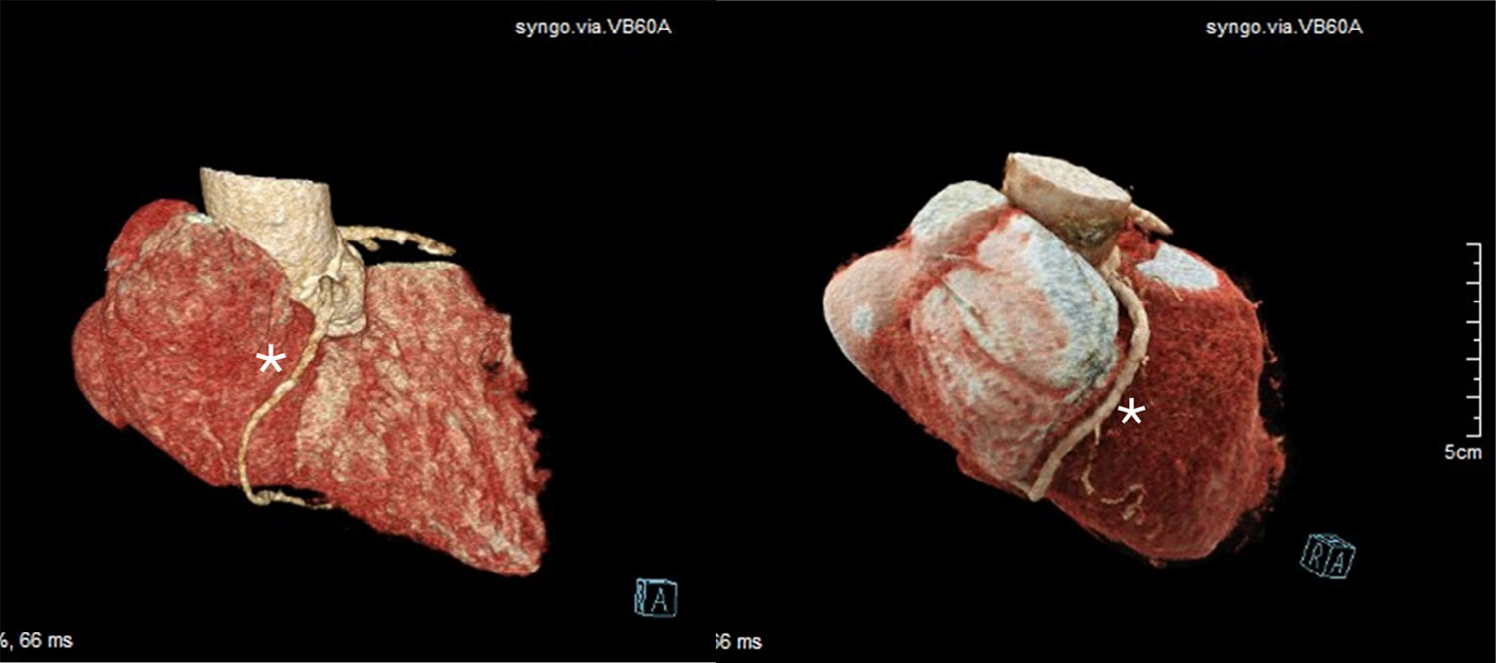
Rendering of the right heart and right atrium with the RCA (*) running in the atrio‐ventricular groove. RCA indicates right coronary artery.

**FIGURE 10:**
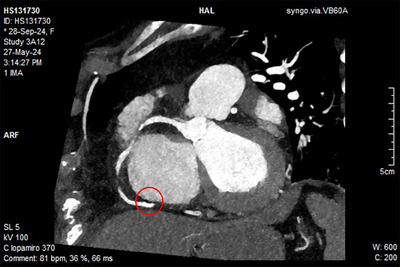
Short axis view of the RCA displaying its entire cursus. Red circle indicating the posterior part of the annulus where the distance between RCA and the anulus can shorten up to 1 mm. RCA indicates right coronary artery.

Most importantly, CT is mandatory to evaluate the anulus and the RCA anatomy prior to transcatheter annuloplasty procedures (Figure 8) due to the close proximity of these two structures, that can be less than 2 mm in almost one‐third of patients with significant TR, and the high risk of RCA impingement (Figures 9 and 10) [37, 79, 80].

Cardiac CT is crucial for TTVR as the Evoque system has the anchors that are deployed between the annular plane and the papillary muscle heads, therefore, an accurate measurement of papillary muscle height is mandatory [81].

New tools such as artificial intelligence and its derivatives, are beginning to show promising results in the field of TTVI. Mattig et al. [82] applied a deep learning algorithm to analyze pre‐interventional CT scans of patients undergoing TTVI (including percutaneous annuloplasty and T‐TEER) and found that lower tenting height was the strongest predictor of significant postprocedural TR reduction. They suggested a threshold of 6.8 mm for T‐TEER patients and 9.2 mm for percutaneous annuloplasty patients.

Lastly, cardiac CT allows the selection of optimal fluoroscopic viewing angles at the preprocedural evaluation [83].

### 6.4 Intracardiac Echocardiography (ICE)

ICE is often used as an imaging method in transcatheter procedures. The ICE catheter has an introducer sheath in the femoral vein and is advanced to the right heart chambers, or, through a transseptal puncture, to the left heart structures.

Typically, transcatheter procedures are performed under TOE guidance, but TOE has several limitations. First, the risk of esophageal injury during prolonged procedures is not negligible. In a prospective study by Freitas‐Ferraz et al. [84] an esophagogastroduodenoscopy performed before and after a TOE‐guided structural cardiac intervention revealed new esophageal lesions in 86% of cases, with 40% classified as “complex” (e.g., intramural hematoma and mucosal laceration). Additionally, TOE image quality can be compromised by shadowing artifacts from the delivery system, intracardiac devices, or anatomical abnormalities [85].

For these reasons, ICE is increasingly being used as an imaging modality in structural interventions.

In the context of TTVI since the TV is often too large for optimal temporal and spatial resolution, ICE is primarily employed as an *adjunctive* imaging technique [86, 87, 88].

When advanced through the right heart to its “home” position, the ICE catheter can provide visualization of the RV inflow‐outflow [85]. With biplane imaging, it aids in device orientation and leaflet grasping, reducing the risk of single‐leaflet grasping [89]. More recently, the “probe in valve” technique has been developed, which involves positioning the ICE probe in the TV commissures by manipulating the steerable catheter [90].

Contraindications for ICE use are intracardiac thrombosis, inferior vena cava occlusion, deep vein thrombosis, and excessive bleeding risk [91].

### 6.5 Cardiac Magnetic Resonance (CMR)

CMR does not have as many applications in TTVI as cardiac CT, at least for the technical aspects of the procedure. This technique is useful to select the right patient as it is the gold standard in the evaluation of RV volume and function [75, 92, 93]. Moreover, CMR can be helpful in quantifying TR especially when the patient has suboptimal echocardiographic views [94]. Cut off for severity of TR are not yet well established in CMR but a prospective observational study of more than 500 patients found that a TR regurgitant volume of 45 mL and a TR regurgitant fraction of 50% is an independent predictor of higher mortality and worse prognosis in patient with FTR [95]. Nevertheless, so far, strong data on TR quantification with CMR as a predictor of postintervention success are lacking, further studies are needed to augment the clinical use of this powerful tool.

## 7 Conclusions

Multimodality imaging is the cornerstone upon which TTVI is built nowadays and provides broad applications of various techniques. The integration of different imaging modalities (echo, CT, ICE fluoroscopy, and CMR) is crucial for procedural success and collaboration among medical professionals (imaging cardiologists, interventional cardiologists, cardiac surgeons, and cardiac radiologists) is mandatory for optimizing patients’ outcomes while reducing complications.

## Data Availability

Data sharing is not applicable to this article as no new data were created or analyzed in this study.
